# Next-generation sequencing impact on cancer care: applications, challenges, and future directions

**DOI:** 10.3389/fgene.2024.1420190

**Published:** 2024-07-09

**Authors:** Mariano Zalis, Gilson Gabriel Viana Veloso, Pedro Nazareth Aguiar Jr., Nathalia Gimenes, Marina Xavier Reis, Silvio Matsas, Carlos Gil Ferreira

**Affiliations:** ^1^ Oncoclínicas&Co/MedSir, Rio de Janeiro, Brazil; ^2^ Medical School of the Federal University of Rio de Janeiro, Rio de Janeiro, Brazil; ^3^ Santa Casa de Misericórdia de Belo Horizonte, Belo Horizonte, Brazil; ^4^ Faculdade de Medicina do ABC, Santo André, Brazil; ^5^ Centro de Estudos e Pesquisas de Hematologia e Oncologia (CEPHO), Sao Paulo, Brazil

**Keywords:** precision medicine, next-generation sequencing, cancer, medical technology, health technology assessment (HTA)

## Abstract

Fundamentally precision oncology illustrates the path in which molecular profiling of tumors can illuminate their biological behavior, diversity, and likely outcomes by identifying distinct genetic mutations, protein levels, and other biomarkers that underpin cancer progression. Next-generation sequencing became an indispensable diagnostic tool for diagnosis and treatment guidance in current clinical practice. Nowadays, tissue analysis benefits from further support through methods like comprehensive genomic profiling and liquid biopsies. However, precision medicine in the field of oncology presents specific hurdles, such as the cost-benefit balance and widespread accessibility, particularly in countries with low- and middle-income. A key issue is how to effectively extend next-generation sequencing to all cancer patients, thus empowering treatment decision-making. Concerns also extend to the quality and preservation of tissue samples, as well as the evaluation of health technologies. Moreover, as technology advances, novel next-generation sequencing assessments are being developed, including the study of Fragmentomics. Therefore, our objective was to delineate the primary uses of next-generation sequencing, discussing its’ applications, limitations, and prospective paths forward in Oncology.

## Introduction

The main fundament of precision oncology is the detailed molecular profiling of tumors to identify specific genetic alterations, protein expressions, and other biomarkers that drive cancer growth (Satam et al., 2023), besides predicting tumors’ biological behavior, heterogeneity, and prognosis (Nakagawa and Fujita, 2018).

DNA and RNA sequencing has rapidly evolved over the past four decades and had two breakthrough moments, first with Sanger sequencing and second with next-generation sequencing (NGS) (Satam et al., 2023). The latter allows broader analyses from fragments of the human DNA, with an extended spectrum of gene sequencing, and captures multiple mutations in a short period (Qin, 2019).

Studies on genome-wide analyses, like The Cancer Genome Atlas (TCGA) and International Cancer Genome Consortium (ICGC) performed with NGS technology, provided comprehensive mutational data (Nakagawa and Fujita, 2018). Such advanced technology does not limit itself to a single tumor site and may be applied to Non-Small Lung Cell Cancer (NSLCC) (de Oliveira Cavagna et al., 2023; Kang et al., 2024), Colon Cancer (Zhu L. et al., 2023; Bachet et al., 2023; Quintanilha et al., 2023), and Melanoma (King et al., 2023; Perez-Perez et al., 2023; Dedeilia et al., 2024) care.

NGS became an indispensable diagnostic tool, and with the increase of genomic profiling in current clinical practice for both diagnosis and treatment guidance, cancer management was reshaped by precision oncology (Mateo et al., 2022). Nonetheless, precision medicine in oncology poses unique challenges (Chan et al., 2021), such as cost-effectiveness and accessibility (Chan et al., 2021; Mateo et al., 2022), with major concerns on how NGS can be properly implemented for all cancer patients, empowering treatments’ decision-making (Mateo et al., 2022). Therefore, we aimed to scope the main applications of NGS, covering its’ applications, pitfalls, and future directions in Oncology.

## Next-generation sequencing: a new standard in cancer management

NGS provides simultaneous sequencing and comprises a platform that uses sequencing-by-synthesis methods such as Illumina (reversible dye terminators) and Ion Torrent (hydrogen ion released) (Satam et al., 2023). The vast amount of NGS data requires advanced bioinformatics tools to properly analyze and interpret each variant and its clinical significance (Qin, 2019).

NGS enables detailed analysis of genetic material from various sources. Among the most used nucleic acids in NGS are genomic DNA (gDNA), RNA, cell-free DNA (cfDNA), and circulating tumor DNA (ctDNA). Each of these nucleic acids offers unique advantages and poses specific challenges for sequencing applications.

NGS marked a substantial advancement in personalized medicine by facilitating the identification of somatic driver mutations, resistance mechanisms, quantification of mutational burden, and germline mutations, laying the groundwork for a novel approach to cancer treatment (Salvo et al., 2021). Multiple studies have shown that mutation analysis can aid clinicians in more accurately classifying tumors and recommending appropriate treatment regimens for patients (Del Re et al., 2024). Such stratification led to the development of a personalized treatment approach, i.e., with the viability of choosing the proper treatment, whether the choice is chemotherapy, targeted therapy, and/or immunotherapy.

### Current perspectives and clinical application

#### Comprehensive genomic profiling on cancer

Comprehensive Genomic Profiling (CGP) enhanced the field of molecular diagnostics, leveraging NGS technologies to analyze a broad array of genetic alterations across a multitude of genes in a single, efficient test (Pankiw et al., 2023; Diks et al., 2024).

CGP offers advantages over traditional methods by requiring smaller tissue samples and reducing the time needed to test for various biomarkers (Table 1). Improvements to CGP, such as RNA fusion assays and liquid biopsies, extend its capabilities beyond gDNA analyses. These additions are valuable for identifying gene fusions and splicing variants, as well as for augmenting the findings from tissue-based CGP, offering a more complete picture of a tumor’s genetic landscape (Tjota et al., 2024).

**TABLE 1:** Benefits of comprehensive genomic profiling in oncology.

Hallmark	Key aspects
Personalized Medicine	By providing a detailed genetic profile of a tumor, CGP supports the implementation of personalized medicine, where treatments are tailored to the unique characteristics of each patient’s cancer
Improved Treatment Outcomes	Patients who receive therapy based on CGP findings can often experience better outcomes, including higher response rates, longer progression-free survival, and, in some cases, improved overall survival
Cost-Effectiveness	Although initially more expensive than single-gene tests, CGP can be more cost-effective in the long term by identifying the most effective therapy from the outset, reducing the need for trial-and-error treatment approaches
Identification of Resistance Mechanisms	CGP can uncover genetic alterations that confer resistance to certain treatments, guiding clinicians in selecting alternative therapies or combination strategies to overcome resistance
Challenges and Considerations on CGP	Despite its advantages, CGP faces challenges, including the need for sufficient tumor tissue samples, the complexity of data interpretation, and the requirement for specialized bioinformatics support to analyze and understand the results. Additionally, there are ongoing discussions regarding the reimbursement and cost coverage of CGP tests by healthcare systems and insurance providers

CGP: comprehensive genomic profiling.

Cancer treatment is often complicated by the development of resistance to therapies. CGP can identify genetic changes that confer resistance to specific drugs, allowing oncologists to adjust treatment plans proactively. For example, the detection of a secondary mutation in the epidermal growth factor receptor (*EGFR*) gene in lung cancer patients who initially responded to *EGFR* inhibitors but then relapsed can guide the switch to alternative therapies designed to overcome resistance (Kulda et al., 2023; Rotow et al., 2024).

Nonetheless, the adoption of CGP across all clinical scenarios remains uneven (Kou et al., 2017). Despite these advantages, data indicate that while 44% of such patients in Japan are recommended new forms of therapy following CGP testing, fewer than 10% receive these recommended treatments, leading many to discover that the test does not change their treatment course (Kage et al., 2024).

The future of CGP lies in its integration with other ‘omics’ data (such as proteomics and metabolomics) and clinical information to develop even more sophisticated models of cancer. This integration promises to enhance our understanding of cancer biology, improve the prediction of treatment responses, and identify novel therapeutic targets.

#### Next-generation sequencing and liquid biopsy

The utilization of NGS technologies through liquid biopsy has emerged as a groundbreaking approach in the diagnosis, monitoring, and treatment planning for patients with cancer. These multifaceted aspects are pivotal for advancing personalized cancer therapy (Lin et al., 2021; Ferreira et al., 2023) (Table 2). These broad-spectrum analyses are crucial for identifying actionable mutations that can guide the selection of targeted therapies, making NGS an indispensable tool in the precision oncology toolbox (Del Re et al., 2024; Yi et al., 2024).

**TABLE 2:** Advantages of liquid biopsies in cancer care.

Hallmark	Key aspects
Minimally Invasive	Liquid biopsies offer a non-invasive alternative to surgical tissue biopsies, significantly reducing patient discomfort and risk of complications
Real-time Monitoring	Liquid biopsies enable real-time monitoring of cancer progression and response to treatment, allowing for timely adjustments in therapy
Detection of Minimal Residual Disease	Liquid biopsies can detect minimal residual disease following treatment, providing early warning signs of relapse
Identification of Resistance Mechanisms	Through the detection of genetic mutations that confer resistance to targeted therapies, liquid biopsies can guide the selection of alternative treatments

Liquid biopsy refers to the non-invasive analysis of tumor-derived material, such ctDNA, circulating tumor cells (CTCs), RNA, and exosomes, present in bodily fluids like blood, urine, or cerebrospinal fluid. This approach offers a dynamic snapshot of cancer’s genetic landscape, enabling real-time tumor evolution assessment, resistance mechanisms, and treatment efficacy) (Lin et al., 2021; Pesta et al., 2022; Herreros-Villanueva et al., 2022; Xia et al., 2023). Table 3 summarizes how liquid biopsies and NGS are transforming Oncology care.

**TABLE 3:** Current applications of Next-Generation Sequencing and Liquid Biopsy.

Hallmark	Key aspects
Early Detection and Diagnosis	NGS analyses of ctDNA or CTCs can identify cancer-specific mutations, potentially allowing for the early detection of cancer before clinical symptoms arise or imaging findings become apparent. This application is particularly promising for cancers that lack effective screening methods
Tumor Heterogeneity and Evolution	Liquid biopsies can capture the genetic diversity of tumors, including primary and metastatic sites. NGS analysis provides insights into tumor heterogeneity and evolution, which are crucial for understanding resistance mechanisms and metastatic potential
Treatment Selection	NGS-based liquid biopsies can identify actionable genetic alterations, guiding the selection of targeted therapies. For example, detecting specific mutations in *EGFR* or *ALK* genes in ctDNA from lung cancer patients can direct the use of appropriate tyrosine kinase inhibitors
Monitoring Treatment Response and Disease Progression	Liquid biopsies allow for the dynamic monitoring of tumor burden and response to treatment, offering a more accurate assessment of therapeutic efficacy over time. An increase in ctDNA levels, for instance, may indicate disease progression or relapse, while a decrease suggests a positive response to treatment

NGS: Next-Generation Sequencing. ctDNA: circulating tumor DNA. CTCs: circulating tumor cells. *EGFR*: epidermal growth factor receptor. *ALK*: anaplastic lymphoma kinase.

The variability in ctDNA levels is critical for understanding the utility of ctDNA as a biomarker in cancer management. The differential detection rates of ctDNA may vary across various cancers. ctDNA may be found in more than 75% of patients with advanced stages of pancreatic, colorectal, gastroesophageal, hepatocellular, bladder, ovarian, breast, head & neck cancers, or melanoma (Bettegowda et al., 2014). Conversely, ctDNA might be less frequent (in fewer than 50% of cases) in patients with primary brain, prostate, thyroid, and renal cancers (Bettegowda et al., 2014). In cancers where ctDNA is more readily detected, NGS can be used to identify actionable genetic mutations that can guide targeted therapy decisions (Del Re et al., 2024; Horgan et al., 2024), whereas in cases where ctDNA is less prevalent, advancements in NGS sensitivity are critical for improving detection rates, thereby broadening the utility of ctDNA analyses across a wider range of cancers and stages (Tjota et al., 2024).

The integration of NGS into the domain of liquid biopsy is particularly crucial in evaluating variant allele frequency (VAF) in ctDNA, which has emerged as a promising biomarker with potential clinical applications (Chen and Zhao, 2019).

VAF are important measures of genetic variation that are used in a broad range of tumor assessments, including its’ purity and ploidy, and it can be measured from both genomic (DNA) and transcriptomic (RNA) sequencing data as the encoded and expressed allele frequencies, respectively (Slowinski et al., 2020). VAF can be assessed using either tissue samples or ctDNA isolated from liquid biopsy, and it may distinguish driver from passenger mutations and the potential germline status of genomic alterations (Galant et al., 2024). Thus, by calculating this genomic biomarker, VAF may represent a surrogate for mutation clonality and can act as a tool to evaluate the genomic heterogeneity of tumors (Boscolo Bielo et al., 2023), which is why lately, an extensive amount of research has focused on using VAF in ctDNA analyses in several types of tumors. This metric is valuable because it can provide insights into the tumor burden within the patient, the efficacy of treatment, and the dynamics of tumor evolution and resistance mechanisms (Hallermayr et al., 2023; Harter et al., 2024), as well as early detection of relapse or disease progression (Pairawan et al., 2020). However, the broad clinical application of VAF measurement in liquid biopsy is contingent upon further validation and research since the accuracy of VAF quantification is highly dependent on the NGS technologies employed (Janku et al., 2017; Manca et al., 2022; Menon and Brash, 2023), and lack of biological threshold definition (Boscolo Bielo et al., 2023), which may vary according to each type of tumor.

As research progresses and more clinical trials incorporate VAF and other NGS-derived metrics, the role of liquid biopsy in cancer care is expected to expand further, offering more personalized, dynamic, and effective treatment strategies for patients.

### Pitfalls

#### Tissue sample quality and integrity

The pathway to obtain high-quality, reliable NGS data is full of challenges, notably from the pre-analytical phase until the NGS sequencing itself. The pre-analytical phase is critical as it encompasses all steps from the initial sample collection to the preparation of nucleic acids for sequencing extracted from formalin-fixed paraffin-embedded (FFPE) (Gu et al., 2023; Astier et al., 2024; Hatanaka et al., 2024).

The chosen tissue blocks must represent a substantial portion of the tumor, ensuring that at least 20% of the material is viable for biomolecular analyses (Gaspersic and Videtic Paska, 2020). The integrity of the sample before sequencing is critical. From the moment of collection, factors such as time to fixation, the duration of fixation, and the conditions under which the sample is stored can significantly affect the nucleic acids of the tumor tissue (Gu et al., 2023). For FFPE samples, the formalin fixation process can induce cross-linking between nucleic acids and proteins, leading to fragmentation and other modifications that challenge the extraction and subsequent analysis processes (Bhagwate et al., 2019), potentially compromising the quality of PCR amplification reactions (Gaspersic and Videtic Paska, 2020).

For instance, the nucleic acid fragmentation and hydrolytic deamination of cytosine can lead to deoxyuridine (dU) and T mismatches, and, eventually, artificial C>T substitutions (Haile et al., 2019; Parker et al., 2019). These artifacts, exacerbated by suboptimal fixation and extraction processes, pose challenges for accurately identifying subclonal driver mutations and other clinically relevant variants. Experimental strategies, such as using uracil-DNA glycosylase and high-fidelity polymerase, and bioinformatic approaches like the Genome Analysis ToolKit (GATK) FFPE filter, offer partial solutions (Bewicke-Copley et al., 2019). Recently Heo et al. (Heo et al., 2024) developed DEEPOMICS FFPE, a deep neural network-based tool trained on paired FF and FFPE sequencing data to distinguish true variants from FFPE-induced artifacts, demonstrating superior performance in preserving true variants while eliminating artifacts.

The DNA Integrity Number (DIN) (Hiramatsu et al., 2023) is an essential metric for assessing the quality of DNA, particularly for NGS applications. It quantitatively evaluates the degree of degradation in a DNA sample. The Agilent TapeStation system (Hiramatsu et al., 2023) provides a standardized method to measure DIN, offering a straightforward way to gauge whether a sample’s DNA integrity meets the requirements for successful NGS. A high DIN value indicates minimal degradation, suggesting that the DNA is likely to perform well in sequencing applications, whereas a lower DIN signals significant degradation, which could compromise the sequencing results.

RNA sequencing (RNA-seq) provides critical insights into gene expression and regulation, though its’ instability presents significant handling challenges (Wang et al., 2019). RNA-seq can identify differentially expressed genes, novel transcripts, and gene fusions. However, RNA is less stable than DNA and more prone to degradation by RNases, making its extraction and storage more challenging. RNA from FFPE samples is particularly difficult to work with due to cross-linking and fragmentation (Byron et al., 2016), especially in older blocks (which may be too poor for clinical testing) (Next-Generation Sequencing, 2024). Other disadvantages of this method include the turnaround time of approximately 1–3 weeks (complexity and labor intensity of testing has limited the widespread inclusion in (Next-Generation Sequencing, 2024)laboratories), the occurrence of bias and imperfections with short-read length RNA-seq technologies generated in sequencing library preparation and short read assembly, and the containing missing values by the read counts of gene expressions, thus resulting in information loss of specific gene and negative impact on downstream analysis (Hong et al., 2020).

Following DNA/RNA extraction and quality assessment, the next critical step in the NGS workflow is library preparation (Fujii et al., 2020; Szadkowska et al., 2022; Michalska-Falkowska et al., 2023). This process involves fragmenting the nucleic acids, repairing the ends, adding adapters, and sometimes incorporating specific indexes for multiplexing samples (Fujii et al., 2020). The quality and integrity of the input material directly influence the efficiency of these steps and the overall complexity and quality of the final library. Libraries from high-integrity samples will more accurately reflect the genome or transcriptome of interest and are more likely to yield robust, comprehensive sequencing data (Szadkowska et al., 2022).

As for liquid biopsies, including those involving ctDNA from blood and non-blood sources, they are vulnerable to the effects of varying anatomical disease distribution on ctDNA concentrations (Tivey et al., 2022). Some disadvantages of the method include low ctDNA to cfDNA ratio owing to the predominance of clonal hematopoiesis, the fact that only certain cell subtypes might release ctDNA into the circulation/low tumor burden, the poor representation of CNS disease, and the poor representation of some tumors (such as early stage non-small-cell lung cancers and sarcomas) (Nikanjam et al., 2022; Tivey et al., 2022). Also, not all detectable cfDNA alterations are cancer-related (Nikanjam et al., 2022). For instance, clonal hematopoiesis is common in patients with cancer and especially in those of advancing age or who previously received radiotherapy (Nikanjam et al., 2022; Tivey et al., 2022), and this feature can confound genomic analysis, especially the specificity of plasma ctDNA (Tivey et al., 2022). Moreover, the half-life of ctDNA is relatively short (approximately 2 h), indicating a need for rapid processing, and without proper preservation and stabilization tubes, the sensitivity of the method may be compromised (Tivey et al., 2022).

#### Cost and accessibility, especially in low and middle-income countries

NGS has seen a remarkable reduction in costs over the years, making it more accessible to researchers and clinicians worldwide (Table 4). In the early 2000 s, the cost of sequencing a human genome was approximately $100 million. However, due to technological advancements and economies of scale, the cost has plummeted to less than $1,000 as of 2022 (The cost of sequencing a human genome, 2021). This dramatic reduction in cost has democratized genomic sequencing, enabling its widespread use in research and clinical settings.

**TABLE 4:** Costs of Next-Generation Sequencing machines and it is panels.

General overview – machines	Cost^a^
Benchtop Sequencers	Entry-level benchtop sequencers are more affordable, typically ranging from $50,000 to $200,000. Examples include Illumina’s MiSeq and Thermo Fisher Scientific’s Ion Torrent series
Mid-Range Sequencers	Mid-range sequencers offer higher throughput and capabilities, with prices ranging from $200,000 to $750,000. Examples include Illumina’s NextSeq and NovaSeq systems
High-End Sequencers	High-end sequencers, such as Illumina’s HiSeq and Pacific Biosciences’ (PacBio) Sequel systems, offer the highest throughput and performance but come with a higher price tag. These systems can cost upwards of $1 million to several million dollars
Nanopore Sequencers	Nanopore sequencers, like Oxford Nanopore Technologies’ MinION and PromethION, offer portable and real-time sequencing capabilities at a relatively lower cost compared to traditional NGS platforms. Prices for nanopore sequencers range from a few thousand dollars to over $1 million, depending on the model and configuration

Panel size and technicalities	Cost^a^
Larger genomic panels – covering a broader range of genes	Between $300 and $1,500 per sample
Deeper sequencing – enhances the detection of rare mutations	Additional $100–$500 per sample
Sample Throughput – batch processing multiple samples simultaneously can reduce the cost per sample	Range from $200 to $800 per sample
Bioinformatics Analysis	Additional $100–$500 per sample
Quality Control	Additional $50–$200 per sample
Overall Cost	Range from approximately $1,250–$5,000 per sample

^a^ Internal source of costs.

Despite these advancements, the cost of NGS testing in low and middle-income countries (LMICs) can still be prohibitive, especially for patients whose costs are out-of-pocket. A study by Schluckebier et al. (Schluckebier et al., 2020) found that the cost of NGS testing in Brazil was significantly higher than other diagnostic modalities in a cohort of advanced lung cancer. The study reported that the average incremental cost of NGS testing was approximately $3,500, which was unaffordable for many patients considering that the average income is about $1,738/monthly (Schluckebier et al., 2020).

Moreover, the continuous advancements in NGS technology and gene coverage add layers of complexity to assessing its’ true cost-effectiveness. These evolutions not only enhance the diagnostic capabilities but also increases the added value, thereby making it difficult to establish a reliable reference cost to be assessed over time. Furthermore, NGS decreasing costs may not be enough to improve access to Precision Oncology, it is important to consider the financial unaffordability of targeted agents (Rivera-Concepcion et al., 2022). A 2012 study assessing NSCLC patients, found an incremental cost of targeted therapy compared to chemotherapy of about $30,000 per QALY (Handorf et al., 2012).

Taken in conjunction, these challenges hamper incorporation of Precision Oncology into healthcare systems (O'Rourke et al., 2020). To address these challenges, efforts are underway to improve access to NGS testing in LMICs. For example, the Global Alliance for Genomics and Health (GA4GH) is working to develop guidelines and standards for genomic data sharing and analysis, with a focus on LMICs (Rehm et al., 2021). Additionally, initiatives such as the Human Heredity and Health in Africa (H3Africa) aim to build genomic research capacity in Africa and improve access to genomic testing (Mulder et al., 2018).

#### Next-generation sequencing and health technology assessment

Health technology assessment (HTA) involves the systematic evaluation of the properties and impacts of health technologies and interventions, including their direct and indirect effects on health outcomes, their costs, and resource utilization (O'Rourke et al., 2020). Moreover, HTA plays a crucial role in incorporation decision-making on a national level (O'Rourke et al., 2020).

While there is no standardized pharmacoeconomic tool for personalized medicine in oncology, the number of studies assessing the cost-effectiveness of NGS analyses has notably increased. Between 2005 and 2007, only three studies were conducted, but from 2014 to 2016, this number rose to 26 (Weymann et al., 2018). Curiously, most studies (76%) utilized the traditional Markov model methodology to evaluate the cost-effectiveness of NGS (Weymann et al., 2018). Furthermore, a significant portion (67%) explored the use of NGS as a risk stratification or prognostic predictor, particularly in breast cancer (44%), employing a health technology assessment modeling typically applied in therapeutic intervention evaluations (Weymann et al., 2018). In terms of NGS-driven targeted therapy, only two studies tackled this subject, both of which surpassed the cost-effectiveness threshold (Djalalov et al., 2014; Doble et al., 2017).

A Brazilian study compared the cost-effectiveness of NGS to sequential single gene testing and discovered that while the molecular diagnosis of patients with advanced NSCLC led to a higher number of true positive genomic alterations, the technology’s cost-effectiveness ratio exceeded the threshold for the Brazilian supplementary healthcare system perspective (Schluckebier et al., 2020). It is important to note that this study not only factored in genomic testing costs but also drug acquisition expenses over a lifetime horizon. Additionally, the authors relied on clinical trial data to estimate treatment duration and the outcomes of each therapeutic agent.

Another limitation of Precision Oncology HTA is the fact that the evidence supporting direct targetable therapies, particularly for rare mutations, is derived from studies with unconventional non-randomized designs, making it challenging to conduct cost-effectiveness analyses on these technologies (Faulkner et al., 2012).

The lack of frameworks for funding and reimbursement related to NGS also led to wide variation in the way it has been incorporated into national healthcare systems. National investment plans or dictation by law guide NGS integration in some countries, while others have a complete lack of plans, policies and governance dedicated to the promotion of NGS (Horgan et al., 2023). This underscores the presence of global disparities surrounding this topic. Efforts are underway to create an HTA framework that aims to facilitate NGS decision-making (Horgan et al., 2023). It is crucial to appreciate regional challenges to have a better understanding of possibilities surrounding NGS expansion and to create opportunities for that end, involving the various stakeholders in the process. In that context, new frameworks will be of great importance.

### Future directions

#### Next-generation sequencing and fragmentomics

Fragmentomics is an emerging field of study focused on analyzing fragments of DNA, RNA, and other molecules shed by cells into bodily fluids like blood (Medina et al., 2023). This approach is gaining traction in oncology due to its’ potential to provide insights into the molecular characteristics of diseases, including cancer, without the need for invasive biopsies (Mathios et al., 2021; Leal et al., 2023).

The development of technologies and computational tools like Fragle, which quantifies ctDNA levels based on cfDNA fragment length distribution, exemplifies the advancements in fragmentomics. These technologies enable the detection and quantification of ctDNA with high sensitivity and specificity, facilitating early cancer detection, the monitoring of disease progression, and the assessment of treatment response. As fragmentomics continues to evolve, it promises to revolutionize personalized medicine by offering more detailed, dynamic, and non-invasive insights into several diseases, thereby improving patients’ outcomes through tailored therapeutic approaches (Zhu G. et al., 2023).

#### Next-generation sequencing and new platforms

The landscape of NGS for cancer research and care is rapidly evolving, with new platforms and approaches emerging to address the complexities of tumor genetics and improve patient outcomes. These innovations are not only enhancing the accuracy and efficiency of genomic sequencing but are also paving the way for more personalized and dynamic cancer treatments. Table 5 exemplifies some of the cutting-edge platforms and approaches in NGS for cancer.

**TABLE 5:** Novel platforms and technologies for Next-Generation Sequencing in Oncology.

Platform/Technology	Key aspects
Single-Cell Sequencing (Ishida et al., 2024)	Single-cell sequencing allows analyses of genetic material from individual cells within a tumor. This approach uncovers the heterogeneity within tumors, providing insights into the mechanisms of cancer evolution, metastasis, and resistance to therapy. By understanding the genetic diversity within tumors at the single-cell level, more targeted and effective therapies can be developed
Long-Read Sequencing Technologies (Yahya et al., 2023)	While traditional NGS technologies generate short reads that can be challenging to assemble in highly repetitive or complex regions of the genome, long-read sequencing technologies, such as those offered by Pacific Biosciences and Oxford Nanopore, produce much longer reads. This ability enhances the detection of structural variants, fusion genes, and complex rearrangements that play critical roles in cancer development and progression, improving the accuracy of genomic analysis
Integrated Multi-omics Platforms (Aldea et al., 2023; Volpe et al., 2023)	Emerging NGS platforms are increasingly integrating genomic sequencing with other ‘omics’ analyses, such as transcriptomics, proteomics, and metabolomics. This integrated approach provides a more comprehensive view of the molecular drivers of cancer, enabling the identification of novel therapeutic targets and biomarkers for treatment response and resistance
CRISPR-Cas9 Based Targeted Sequencing (Malekshoar et al., 2023)	The integration of CRISPR-Cas9 genome editing technology with NGS allows for targeted sequencing of specific genomic regions of interest, enhancing the efficiency and specificity of sequencing cancer-related genes, and enabling the identification of mutations and alterations with greater precision. It holds promise for the development of highly targeted diagnostic tests and the discovery of new therapeutic targets
Artificial Intelligence and Machine Learning-Enhanced Analysis (Thirunavukkarasu et al., 2024)	The application AI and machine learning algorithms to NGS data is transforming the analysis and interpretation of complex genomic datasets. These technologies can identify patterns and predictive markers within large-scale genomic data that may not be apparent to human analysts, developing predictive models for cancer prognosis, treatment response, and the identification of novel therapeutic targets
Portable and Real-time Sequencing Devices (Glowienka-Stodolak et al., 2024)	The development of portable NGS devices, such as the MinION from Oxford Nanopore, enables real-time genomic sequencing in clinical settings, research laboratories, and even in field conditions. This accessibility could revolutionize cancer diagnostics and monitoring, allowing for immediate genomic analysis and decision-making regarding treatment strategies
Digital Spatial Profiling (Glyn et al., 2024; Su et al., 2024)	Digital spatial profiling is an innovative approach that combines NGS with *in situ* analysis of protein and RNA biomarkers within the tumor microenvironment. This technology provides spatial context to genomic data, enabling the understanding of the tumor architecture and the interaction between cancer cells and the immune system, which is vital for the development of effective immunotherapies

NGS: Next-Generation Sequencing. AI: artificial intelligence.

#### Improving access to precision medicine in oncology

NGS became increasingly integrated into clinical practice, and it is crucial to assess its cost-effectiveness accurately. Traditional cost-effectiveness analyses often rely on clinical trial data, which may not fully capture the real-world effectiveness and cost implications of NGS testing (Klonoff, 2020). Based on that, future studies should incorporate real-world evidence (RWE) to provide a more comprehensive understanding of the value of NGS and Precision Oncology. By leveraging RWE, researchers can evaluate the long-term effectiveness, safety, and cost-effectiveness of NGS testing in real-world settings. This approach allows for a more accurate assessment of the value of NGS and can inform decision-making regarding its adoption and reimbursement.

## Final comments

NGS testing has improved precision medicine and Oncology care. Although major improvements in technology, applicability and costs have been already addressed, NGS still may relays as an idealistic medical tool instead of a broader realistic instrument for cancer management.
